# Apropos of Plastic Fillings

**Published:** 1880-05

**Authors:** J. Allen Osmun


### CORRESPONDENCE.

Apropos of Plastic Fillings.—An incident came under my notice to-day that may interest some readers of The Independent Practitioner. About six years ago I was filling teeth for a gentleman and had prepared two proximal cavities for the gold, but daylight being scarce, put some cotton saturated with sandrach varnish into the cavities, and made an appointment for a sitting, about ten days later. The gentleman was suddenly called away and had not time to have them filled before going. To-day he came in the office, and made the remark, "Don't you think it about time these teeth were filled?" and said that he had been West and just got back, and as those teeth had never troubled him he had done nothing to them. On making an examination I found the teeth all right, except a little decay around the margins, the inside of cavities presenting a perfectly white and hard surface.

It only goes to show how sometimes plastic fillings surprise us in their lasting qualities. The fluids of the oral cavity must have been perfectly normal. The cotton was as hard as gutta percha, and could detect no odor except from outside piece, and only a little from it. I do not, however, recommend cotton and sandrach as a *permanent filling*.

J. ALLEN OSMUN.

